# Synthesis of a Novel, Biocompatible and Bacteriostatic Borosiloxane Composition with Silver Oxide Nanoparticles

**DOI:** 10.3390/ma15020527

**Published:** 2022-01-11

**Authors:** Denis N. Chausov, Veronika V. Smirnova, Dmitriy E. Burmistrov, Ruslan M. Sarimov, Alexander D. Kurilov, Maxim E. Astashev, Oleg V. Uvarov, Mikhail V. Dubinin, Valery A. Kozlov, Maria V. Vedunova, Maksim B. Rebezov, Anastasia A. Semenova, Andrey B. Lisitsyn, Sergey V. Gudkov

**Affiliations:** 1 Prokhorov General Physics Institute of the Russian Academy of Sciences, 119991 Moscow, Russia; d.chausov@yandex.ru (D.N.C.); veronausckova@mail.ru (V.V.S.); dmitriiburmistroff@gmail.com (D.E.B.); rusa@kapella.gpi.ru (R.M.S.); ad.kurilov@gmail.com (A.D.K.); astashev@yandex.ru (M.E.A.); uvarov@kapella.gpi.ru (O.V.U.); v.kozlov@hotmail.com (V.A.K.); mvedunova@yandex.ru (M.V.V.); rebezov@ya.ru (M.B.R.); 2 Mari State University, 424000 Yoshkar-Ola, Russia; dubinin1989@gmail.com; 3 Bauman Moscow State Technical University, 105005 Moscow, Russia; 4 The Institute of Biology and Biomedicine, Lobachevsky State University of Nizhny Novgorod, 603105 Nizhny Novgorod, Russia; 5 V.M. Gorbatov Federal Research Center for Food Systems, Russian Academy of Sciences, 109316 Moscow, Russia; semmm@mail.ru (A.A.S.); info@fncps.ru (A.B.L.)

**Keywords:** borosiloxane, composite, silver oxide, nanoparticles, antibacterial, cytotoxicity, biocompatibility

## Abstract

Microbial antibiotic resistance is an important global world health problem. Recently, an interest in nanoparticles (NPs) of silver oxides as compounds with antibacterial potential has significantly increased. From a practical point of view, composites of silver oxide NPs and biocompatible material are of interest. A borosiloxane (BS) can be used as one such material. A composite material combining BS and silver oxide NPs has been synthesized. Composites containing BS have adjustable viscoelastic properties. The silver oxide NPs synthesized by laser ablation have a size of ~65 nm (half-width 60 nm) and an elemental composition of Ag_2_O. The synthesized material exhibits strong bacteriostatic properties against *E. coli* at a concentration of nanoparticles of silver oxide more than 0.01%. The bacteriostatic effect depends on the silver oxide NPs concentration in the matrix. The BS/silver oxide NPs have no cytotoxic effect on a eukaryotic cell culture when the concentration of nanoparticles of silver oxide is less than 0.1%. The use of the resulting composite based on BS and silver oxide NPs as a reusable dry disinfectant is due to its low toxicity and bacteriostatic activity and its characteristics are not inferior to the medical alloy nitinol.

## 1. Introduction

Currently, there is a significant growth of interest in metal oxide nanoparticles (Me_x_O_y_NPs) as compounds with antibacterial potential, including Ag [1,2], which find many applications in technology and medicine. Despite the large number of antibiotics available for treatment, resistance to almost all of them has been confirmed. Antibiotic resistance may occur soon after the approval of a new drug for use [3,4]. The mode of antibacterial action for metals includes disruption of enzyme functioning [5], reactive oxygen species (ROS) production (Fenton’s reaction) [6], disruption of cell membrane functioning, prevention of absorption of essential microelements by microorganisms [7] and genotoxic action [8,9]. The issues of compatibility of the polymer matrix and nanoparticles are raised in articles [10,11,12,13], which are relevant in applied aspects. Controlled synthesis of a polymer in the presence of inorganic NPs permits the generation of a blend with desired physicochemical properties. Distribution of silver NPs in a polymer matrix increases the effectiveness of antibacterial action via controlled release of Ag^+^ cations which can significantly reduce the transmission of infectious agents [14,15,16,17,18]. The synthesis of a composition of silver oxide NPs and a polymer also prevents particle aggregation, because Ag^+^ cations are well distributed in the matrix [19]. In addition, a polymer doped with silver NPs prevents the reproduction and development of microorganisms on the surface [20,21]. Borosiloxane (BS) is a polymeric material with unique physical properties. It is a characteristics material with needed stickiness [22], self-repair [23,24] and crack resist ones [25]. Therefore, BS can be a used in development of composites with NPs. BS-based materials are widely used in various industries. An example of BS is used in manufacturing of protective sport wear for racing, skiing and other extreme sports [26]. Thus, the BS and silver oxide NPs system (with antibacterial properties) can find wide application in the production of sports equipment. The development self-healing electronics based on BS has been reported [27,28,29,30]. BS-based polymers can also be used in the development of electro-optical devices and electronic apparatus [31,32,33]. A nanocomposite based on BS and silver oxide NPs for biomedical applications has been synthesized. A comprehensive study of antibacterial, cytotoxic and mechanical properties of the material has been carried out. A main objective was to reduce the amount of administered dopant while maintaining strong antibacterial properties.

## 2. Materials and Methods

### 2.1. Characterization of Silver Oxide Nanoparticles

Silver oxide NPs were synthesized by laser ablation technique using a pulsed ytterbium-doped fiber laser (YDFL). Laser pulses: wavelength 1064 nm, pulse duration 4–200 ns, frequency of repetition 20 kHz, average power ≤20 W, average energy 1 mJ. Radiation time was 5–20 min. Deionized water was chosen as work liquid. A 10 g of pure Ag (99.9%) was placed in the 10 mL of water so that water covered the all surface of the metal (maximum water layer thickness ≤1 mm. The experimental setup and its main components are described in [34].

NPs size and ζ-potential of NPs were measured with Zetasizer Ultra Red Label (Malvern Panalytical, Malvern, UK). In addition, the diameter of nanoparticles was estimated using an CPS 24,000 disk analytical centrifuge. The NPs size, morphology and elements composition were analyzed by transmission electron microscopy using Libra 200 FE HR (Carl Zeiss, Jena, Germany). The NPs colloid spectra were recorded using Ocean Optics USB3000T spectrometer (Ocean Optics, Dunedin, FL, USA).

### 2.2. BS and NPs Composition Synthesis and Study of Rheological Properties

The BS was synthesized from hydroxyl-terminated polydimethylsiloxane (PDMS) (MW 20 kDa) and boric acid (BA) (99.9% BA, 57.1% boric anhydride, ~75 μm particles size). PDMS was mixed with boric acid in a ratio of 10:1. Mixture of PDMS and BA were heated at temperatures ≥200 °C. BS was dissolved in ethanol and doped by NPs with known concentration: 0.1, 0.01 and 0.001% of mass. The ethanol was then evaporated at reduced pressure. BS without dopants was also dissolved in ethanol and then dried under reduced pressure. The study of the rheological behavior of borosiloxane-based nanocomposites was carried out on a rheometer MCR 302e (Anton Paar, Graz, Austria). The multiparameter rheological equations were used to evaluate the non-Newtonian properties of studied polymers [35]. These equations may be applied in a wide shear ranges.

### 2.3. BS Segments Preparation to Analysis

The synthesized materials were rolled out on a substrate heated to 40 °C. The thickness of resulting polymer film was 0.7–0.9 mm. The polymer film was cut into rectangular pieces with a size of 20–25 mm and a total area of 10 cm^2^. The films were placed in 20 mL of water to further measuring ROS and markers of biomacromolecules damage.

### 2.4. Hydrogen Peroxide Generation Assay

The hydrogen peroxide (H_2_O_2_) concentration was measured by highly sensitive chemiluminescence (CL) method. The method based on oxidation of the luminol-p-iodophenol by horseradish peroxidase with subsequent emission of light quanta. Measures were carried out with ultrasensitive Biotox-7A-USE high sensitivity chemiluminometer (ANO Engineering Center—Ecology, Moscow, Russia). The H_2_O_2_ concentration was evaluated with calibration curves. These curves were built on the CL intensity values of samples with known H_2_O_2_ concentrations. The H_2_O_2_ concentration used for calibration was measured by spectrophotometry using Cintra 4040 (GBC Scientific Equipment, Boulevard, Australia) at a wavelength of 240 nm with a extinction coefficient of 43.6 (M^−1^ × cm^−1^) [36]. The samples were mixed with 1 mL of a “counting solution” in polypropylene vials. “Counting solution” contained 1 mM Tris-HCl buffer (pH 8.5), 50 μM p-iodophenol, 50 μM luminol, 10 nM horseradish peroxidase. The “counting solution” was prepared immediately before a study. The chemiluminescence method sensitivity <1 nM H_2_O_2_ [37].

### 2.5. OH-Radicals Generation Assay

Concentrations of OH-radicals were measured by reaction with coumarin-3-carboxylic acid (CCA). CCA hydroxylated to 7-hydroxycoumarin-3-carboxylic acid (7-OH-CCA). The 7-OH-CCA is a widely used fluorescent probe for detecting of OH-radicals generation. Phosphate buffer (0.2 M, pH 6.8) was added to a CCA solution in water (0.5 mM, pH = 3.6). Experimental (and control) samples were heated for 2 h at 80 ± 0.1 °C. The fluorescence intensity of 7-OH-CCA was measured with a JASCO 8300 spectrofluorimeter (JASCO, Tokyo, Japan) with excitation and emission wavelengths 400 nad 450 nm, respectively. Calibration was carried out with solution of commercial 7-OH-CCA [38].

### 2.6. Long-Lived Reactive Protein Species Generation Assay

The interaction of radicals with other molecules often leads to emission of light quanta. Therefore, chemiluminescence method is a suitable and high sensitive method of measured free radical reactions. Concentrations of long-lived reactive protein species were measured in heated protein solutions by the chemiluminescence with Biotox-7A chemiluminometer (ANO Engineering Center—Ecology, Moscow, Russia). The measurements were carried out at 25 °C in the dark, in 20 mL plastic polypropylene vials for liquid scintillation counting (Beckman, Brea, CA, USA). These modifications increased the sensitivity of method in ~200 times in comparison with canonical conditions (0.1 mL) [39]. The proteins without heating were used as as controls. The more detailed description of the method may be found in [40].

### 2.7. Enzyme-Linked Immunosorbent Assay (ELISA)

DNA (350 μg per mL) were denatured by boiling 5 min with subsequent cooled on ice 3–4 min. 42 μL of each sample were added in a correspond well of 96-wells plate. DNA was immobilized by 3 h incubation for at 80 °C until completely drying of solutions. The blocking of nonspecific adsorption was performed by overnight incubation in 1% skimmed milk solution in 0.15 M Tris-HCl buffer, pH 8.7 supplemented by 0.15 M NaCl at room temperature. Further, 100 μL of blocking solution with anti-8-OG antibody (dilution 1:2000) was added in each well. Plates were incubated 3 h at 37 °C. Wells were washed twice with 50 mM Tris-HCl buffer (pH 8.7) supplemented by 0.15 M NaCl and 0.1% Triton X-100. Further, secondary anti-mouse-IgG antibody conjugated with horseradish peroxidase (dilution 1:1000) in blocking 80 μL of solution was added into each well. Plate was incubated 1.5 h at 37 °C. Further, all wells were washed 3 times as described above. Next, 100 μL of 75 mM pH 4.2 citrate buffer containing 18.2 mM ABTS and H_2_O_2_ (2.6 mM) were added to each well. The chromogenic reactions were stopped by 1.5 mM NaN_3_) upon reaching color. The optical density was evaluated with a photometer (Titertek Multiscan, Helsinki, Finland) at 405 nm. The more detailed information about method may be found in previous work [41,42].

### 2.8. Antibacterial Properties Assay

BS or NPs·BS composite films were preliminary sterilized by three washes with 70% ethanol. Escherichia coli (Gram-negative) was chosen to antibacterial properties assay as most common human commensal. Bacteria were cultured in LB medium by standard protocols. Bacteria concentrations were measured by spectrophotometric study with drop spectrophotometer UV5Nano Excellence (Mettler Toledo, Columbus, OH, USA). In each experiment 10 μL of the medium with known concentration of *E. coli* was diluted in a 1 mL LB medium. This suspension was applied to the surface of BS or NPs·BS composite films (10 × 15 × 0.8 mm^3^), placed in sterile chamber. The samples were incubated in an ES-20 shaker incubator (Biosan, Latvia) at 37 °C and ~150 rpm. After exposition, the concentration of bacteria was measured with a drop spectrometer.

The effects of BS or NPs·BS compositions on detachment of *E. coli* from a substrate were evaluated by the technique described below. *E. coli* were cultured on Petri dishes with LB agar. The Petri dishes were incubated 8 h at 37 °C. Further studies compositions were applied on LB agar in Petri dishes at 1 h. Finally, compositions were removed and concentrations of *E. coli* on compositions surfaces were evaluated by microscopy. Microorganisms stained with crystal violet indicator and counted with microscope Micromed MET (Micromed, Moscow, Russia) at magnification 1000. The experimental details were described in work [43].

### 2.9. Assay of Biocompatibility with Mammalian Cells

The SH-SY5Y human neuroblastoma cell culture was chosen as standard in vitro test system. These cells line widely used to study mammalian cells viability, proliferation, differentiation, morphology and functions [44].

**Figure 1:**
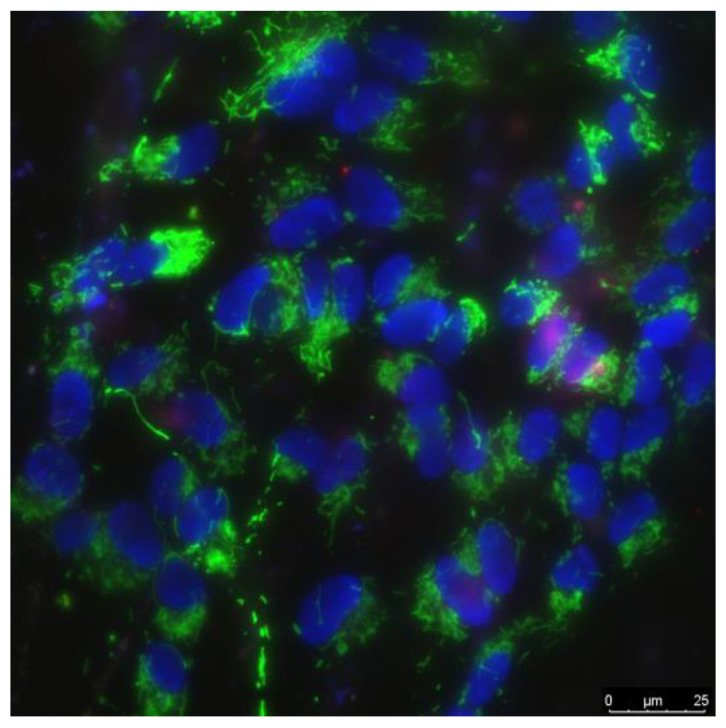
The example of photomicrograph of a cell culture. Green staining corresponds to mitochondria (used to evaluate shape and size of cells); blue staining indicates nuclei of viable. Purple staining indicates of nuclei of non-viable cells.

The cells were cultured by standard protocols in CO_2_ incubator (Binder, Tuttlingen, Germany). Culture medium contained DMEM (Biolot, Moscow, Russia), 10% fetal calf serum (Gibco, Dublin, Ireland) and 30 μg/mL gentamicin. Cells were cultured on surface of BS and NPs·BS compositions samples (20 × 20 mm^2^) in Petri dishes (35 mm diameter) in 3 mL of culture medium. The initial cells count was 10^4^ cells per cm^2^ in each experiment. Cells were cultured on the experimental samples within 3 days. Cells viability was evaluated by fluorescence microscopy. Cells were stained 10 min with fluorescent probes 2 μg/mL Hoechst 33342 (Sigma, Burlington, MA, USA) and 2 μg/mL propidium iodide (Sigma, Burlington, MA, USA) to evaluate the amount of live and dead cells, respectively. An example of cells stained by Hoechst 33342 stains (marks all cells), and propidium iodide (marks dead cells) are shown on Figure 1. Cells were analyzed with Leica DMI6000 confocal microscope (Leica, Wetzlar, Germany). In each sample ≤500 cells were counted for analysis [45].

Cell proliferation was characterized using the mitotic index (MI). MI of cells were evaluated in logarithmic phase of growth (3 days after seeding). MI is a proportion of number of cells in mitosis to number of all living cells in a field of view. MI was evaluated by fluorescence microscopy. Cells were stained with Hoechst 33342 fluorescent dye (Sigma, Burlington, MA, USA). Mitotic cells were identified by the chromatin properties in prophase (P), metaphase (M), anaphase (A), and telophase (T). In each sample ≤500 cells were taken in a further analysis. The mitotic index (MI) was calculated by the formula MI = (P+M+A+T)/N × 100%, where P, M, A and T is the cells amount in prophase, metaphase, anaphase and telophase mitosis stage, respectively, and N is amount of all studied cells [46].

### 2.10. Statistics

Statistical processing was carried out with SigmaPlot, Origin and MS Exel software. Data are presented as means ± standard errors of mean (SEM). In each condition results of least 3 independent experiments were used for statistical processing. Criteria used to evaluation of statistically significant of differences between sample means are indicated in legends of corresponding figures.

## 3. Results and Discussion

**Figure 2:**
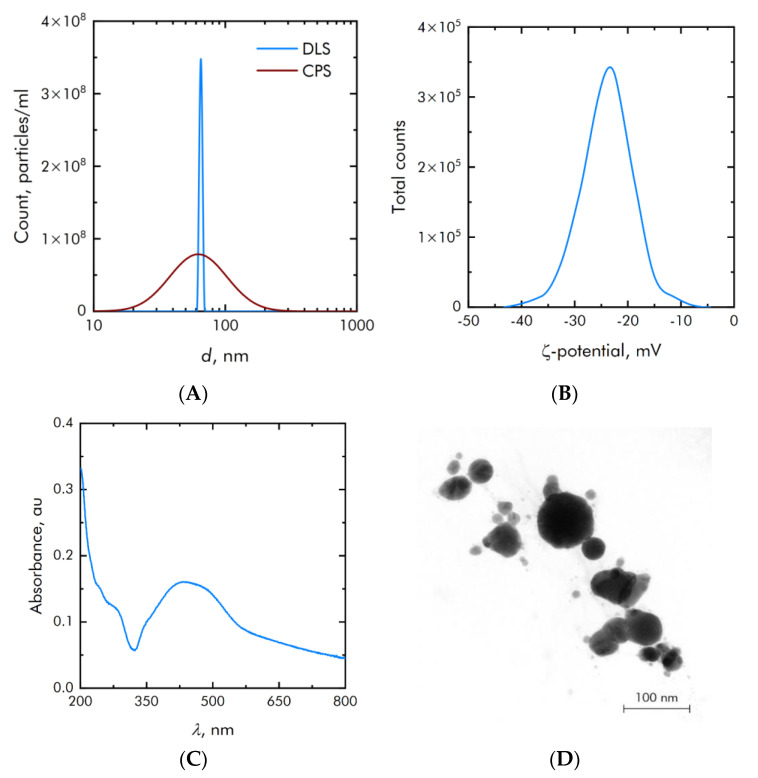
Physicochemical characterization of silver oxide NPs. (**A**)—Concentration (DLS) and size (CPS) distribution of silver oxide NPs. (**B**)—Zeta potential distribution of synthesized silver oxide NPs. (**C**)—Optical absorption spectrum of silver oxide NPs colloid. (**D**)—TEM microphotography silver oxide NPs.

Silver oxide NPs were synthesized by dint of laser ablation technique in aqueous solution. The nanoparticle size distribution was measured by two independent methods: differential centrifugation and dynamic light scattering. The measurement results are shown in Figure 2A. The main mode of the resulting distribution is 65 nm, and the half-width is 60 nm. The concentration of particles in the initial colloid was 3.5 × 10^8^ particles per ml. The zeta potential of the synthesized colloidal particles is in a wide range from −40 to −8 mV with an average value of −22 mV (Figure 2B). Figure 2C shows the optical absorption spectrum of the synthesized colloid, which corresponds to silver oxide nanoparticles.

**Figure 3:**
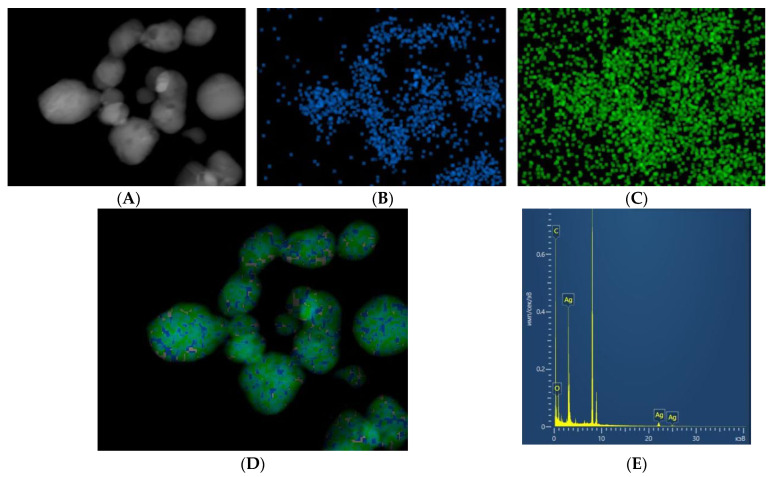
Elemental analysis of the resulting nanoparticles. (**A**)—TEM image of Ag_2_O nanoparticles. (**B**)—Visualization Ag Kα1. (**C**)—Visualization O Kα1. (**D**)—multilayer image (Ag Kα1 + O Kα1). (**E**)—Spectrum of the sample (X-axis—Energy, keV, Y-axis—Distribution, pulse/sec/eV).

Electron microscopy was performed to confirm the data of granulometric and spectral analysis. TEM images of nanoparticles are shown in Figure 2D. In addition to the morphology of nanoparticles, electron microscopy allows for elemental analysis (Figure 3). The results of electron microscopy confirm that the synthesized nanoparticles correspond to silver oxide, and their size is about 80 nm. The ratio of Ag/O atoms corresponds to the oxide Ag_2_O.

**Figure 4:**
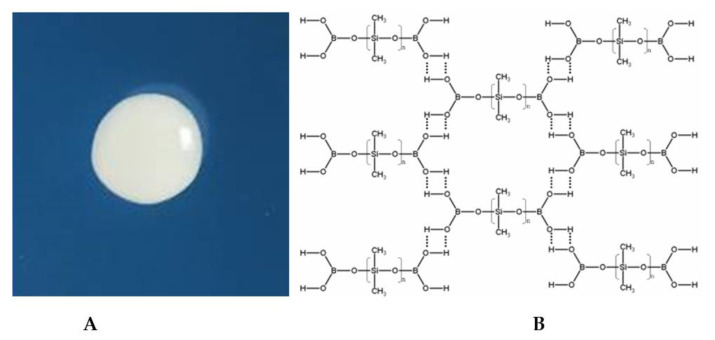
(**A**)—Photo of the BS, (**B**)—representation of the BS structure. Hydrogen bonds are indicated by dotted line.

Borosiloxane polymer material was chosen as the carrier medium for silver oxide nanoparticles. The picture of the borosiloxane sample and its structural formula is shown in Figure 4. The molecular structure of borosiloxane is close to organosilicon compounds containing Si-O and B-O fragments. The mechanical properties of borosiloxane can be widely varied at the synthesis stage. This will depend on the molecular weight of PDMS, temperature conditions, as well as functional additives [47]. In borosiloxane, covalent bonds are not destroyed during any deformations. As a result, borosiloxane can undergo multiple destructions while preserving the properties of the material.

Borosiloxane has a low surface energy, which excludes the possibility of a strong chemical interaction between metal oxide nanoparticles and the polymer matrix [48,49]. Nevertheless, the formation of S-Ag interactions in the presence of aliphatic disulfides in the matrix has been reported [50,51]. Thus, the synthesized materials should be considered as compositions or blends rather than composites.

The high stability of the composition is due to the high viscosity of the polymer matrix, which greatly increases the diffusion time of nanoparticles and almost completely eliminates particle agglomeration. Measurements of rheological and bacteriostatic properties were carried out for 3 months. During this time, no systematic changes were detected in the results obtained.

**Figure 5:**
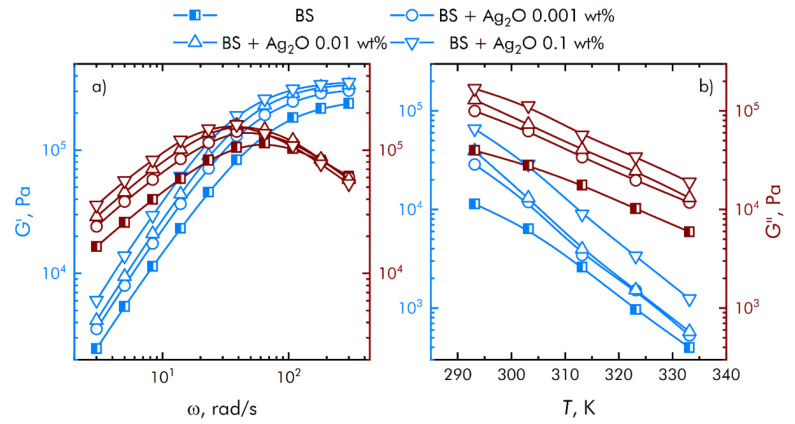
Frequency (**a**) and temperature (**b**) dependences of viscoelastic properties of the resulting compositions.

The results of rheological measurements are shown in Figure 5. The contribution of viscous and elastic properties depends on the frequency of the applied force. The storage modulus *G*’ increases monotonically with increasing oscillation frequency and reaches a constant value in the high frequency region. At the same time, the loss modulus *G*’’ has a characteristic peak in the region of 40–60 rad/s, after which there is a decrease in viscous losses. Thus, the transition region from a viscous state to an elastic one can be adjusted at the synthesis stage by the previously mentioned methods.

Figure 5 also shows the behavior of the rheological properties of the resulting compositions with varying dopant concentrations. An increase in the mass content of the dopant leads to a significant increase in both components of the shear modulus. Synthesized compositions have a complex rheological response depending on the frequency of the applied force due to microstructural rearrangements.

**Figure 6:**
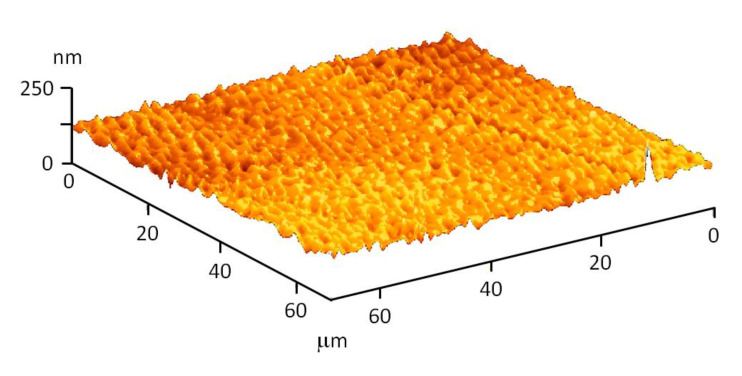
AFM surface topography of a composition based on borosiloxane and silver oxide nanoparticles.

The resulting compositions are homogeneous, which is also confirmed by atomic force microscopy (AFM). Figure 6 shows the topography of the composition surface. It is clearly visible that there are no cracks, breaks and other defects on the surface of the material.

**Figure 7:**
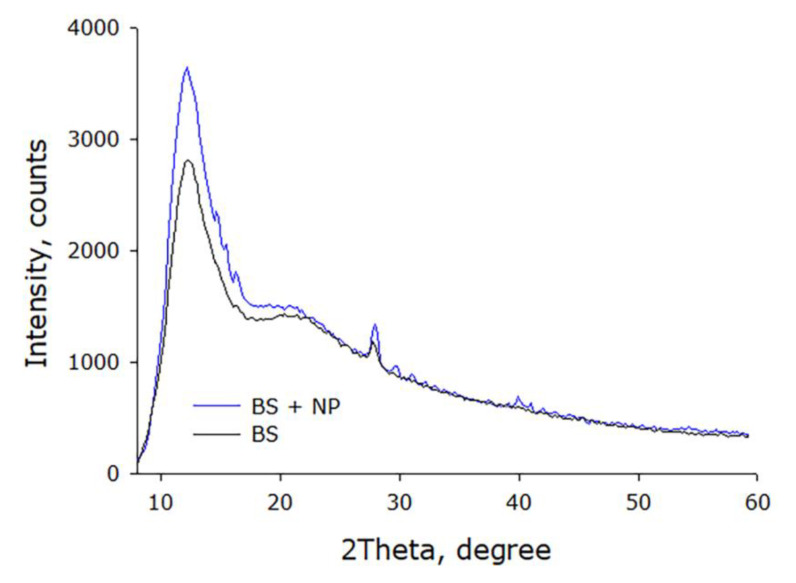
X-ray diffraction patterns of the resulting compositions.

The crystallographic structure of the compositions was studied by X-ray (XRD) diffraction analysis. The X-ray diffraction patterns of the obtained compositions are characterized by the presence of a wide diffraction peak in the range from 5 to 10° (Figure 7). The presence of this peak mainly indicates the amorphous nature of the material. At the same time, the presence of microcrystalline domains with a corresponding peak of 2θ = 12° is observed, confirming the semi-crystalline nature of the material.

**Figure 8:**
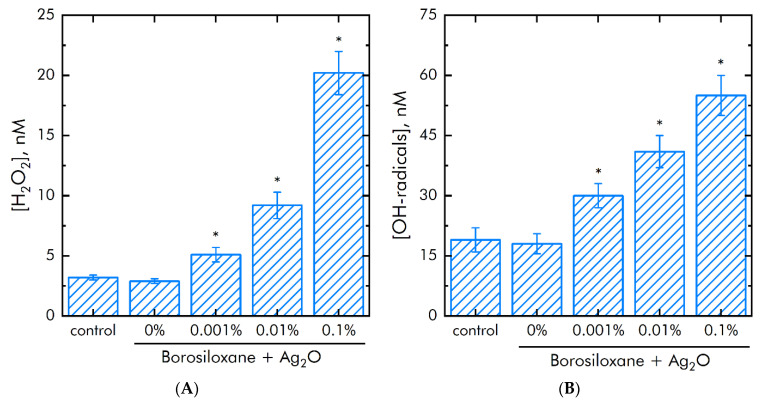
Influense of a BS and composition Ag_2_O NPs·BS on the generation of ROS during 2 h at 40 ℃. (**A**)—H_2_O_2_ generation. (**B**)—OH-radicals production. *—*p* < 0.05 vs. control. Data are shown as means ± SEMs.

Metals with variable valence and their oxides can induce generation of ROS. The effect of BS/silver oxide NPs on ROS production was studied. Hydrogen peroxide (H_2_O_2_) and hydroxyl radicals (OH-radicals) were chosen as representative ROS. First ROS is the most long-lived ROS (Figure 8A). Second ROS is the most reactionary (Figure 8B). It was shown that BS did not change on H_2_O_2_ and OH-radicals concentration in samples. The composition Ag_2_O NPs BS increased H_2_O_2_ concentration on 45, 300 and 700% at mass percentage of Ag_2_O NPs 0.001, 0.01 and 0.1%. The composition Ag_2_O NPs BS increased the OH-radicals concentration in samples by 35% at a NPs concentration of 0.001%. The nanoparticle concentration of 0.01% and 0.1% increased the OH-radicals concentration in samples by 2 and 3 times, respectively.

**Figure 9:**
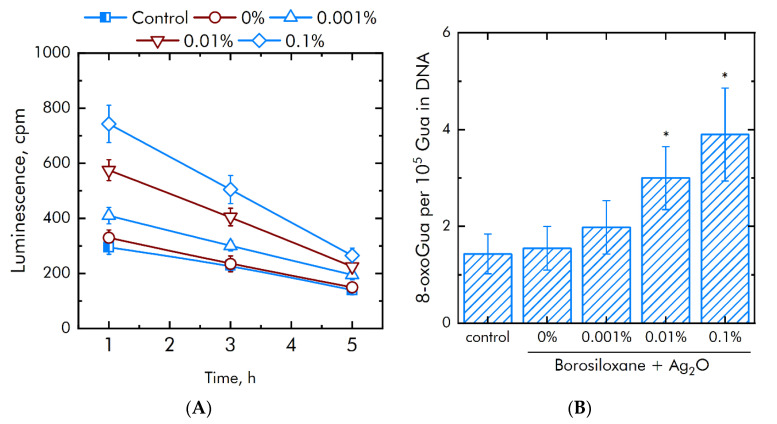
Effect of BS and composition Ag_2_O NPs·BS on markers of proteins and DNA damage generation. (**A**)—Production of long-lived reactive protein species. (**B**)—Production of 8-oxoGua. *—*p* < 0.05 versus control. Data are shown as means ± SEMs.

Generation of high amount of ROS can lead to damage to DNA and proteins. Concentrations long-lived reactive proteins species (LRPS) and 8-oxoguanine (8-oxoGua) were evaluated. First compound is a protein oxidation damage marker. Second compound is DNA damage marker. The effect of a composition Ag_2_O NPs·BS on rate of generation or half-life of LRPS was studied (Figure 9A). BS without NPs did not change generation or half-life of LRPS. Supplementation of Ag_2_O NPs in BS doubled the thickness of the rate LRPS formation. Supplementation of 0.001% Ag_2_O NPs increased LRPS production at 25%. With the Ag_2_O NPs concentration increased to 0.01%, the generation of LRPS increases by 75%, with a concentration of 0.1% by 125%. At the same time, Ag_2_O NPs did not change the half-life of LRPS. The half-lives of LRPS in all samples are same and did not exceeded 5 h.

The effect of a composition Ag_2_O NPs·BS on the 8-oxoGua generation in vitro was studied (Figure 9B). BS did not increase 8-oxoGua generation. The composition Ag_2_O NPs·BS significantly increased a rate of 8-oxoGua generation compared with BS. At Ag_2_O NPs concentrations of 0.001, 0.01 and 0.1%, the production of 8-oxoGua increased by 0.3, 2.1 and 2.5 times, respectively.

**Figure 10:**
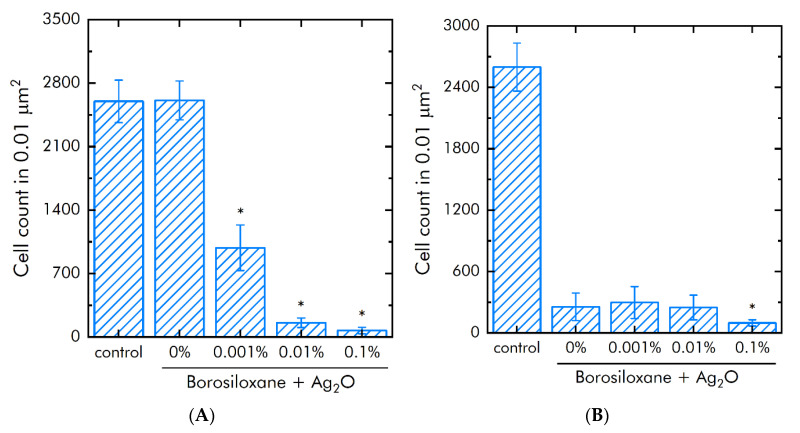
Effect of BS and composition Ag_2_O NPs·BS on the *Escherichia coli* proliferation and adhesion. (**A**)—*E. coli* proliferation during 24 h. (**B**)—Bacteria count on a substrate after detachment. *—*p* < 0.05 vs. control. Data are shown as means ± SEMs.

The influence of Ag_2_O NPs BS and BS on *E. coli* proliferation and adsorption of was studied (Figure 10A). It was shown that BS without Ag_2_O NPs did not change the growth *E. coli* bacteria. Supplementation of Ag_2_O NPs in BS decreased *E. coli* amount on studied materials by 62% at 0.001% Ag_2_O NPs, by 94% at 0.01%Ag_2_O NPs and by 97% at 0.1% Ag_2_O NPs.

The effect of a composition Ag_2_O NPs·BS on *E. coli* bacteria adhesion were investigated (Figure 10B). BS without Ag_2_O NPs decreased adsorption the *E. coli* bacteria from the substrate in 10 times. The supplementation of BS by 0.001 and 0.01% Ag_2_O NPs did not change on adhesion the *E. coli*. Increasing mass concentration of Ag_2_O NPs in the dose to 0.1% decreased bacteria adsorption in 250% times compared with BS. The amount of bacterial cells on the 0.1% Ag_2_O NPs·BS decreased in 27 times compared with control.

**Figure 11:**
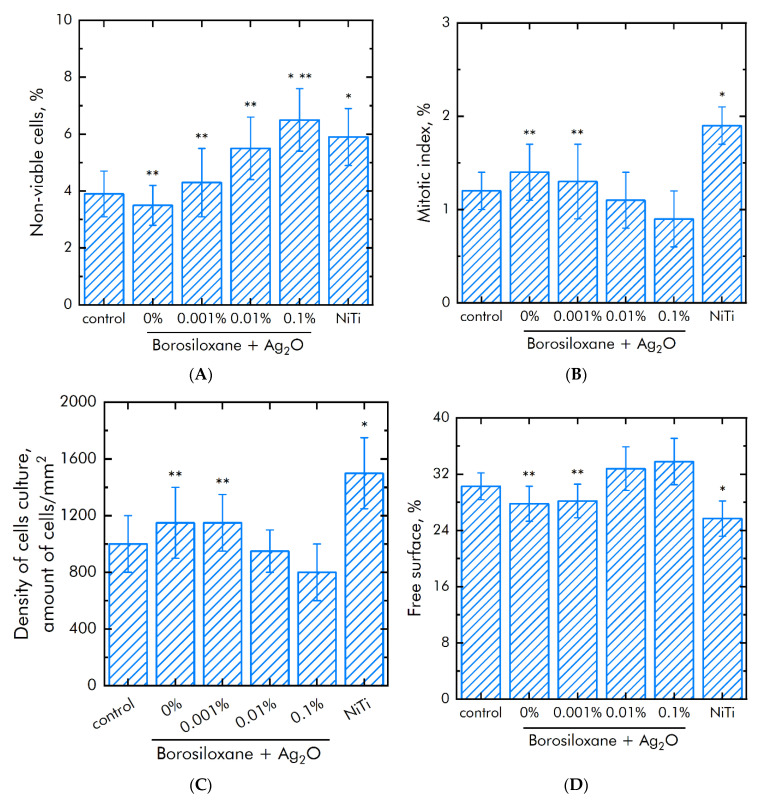
Effect of a BS and composition Ag_2_O NPs·BS the of cell line SH-SY5Y proliferation, adhesion and survival. (**A**)—Survival of cells. (**B**)—Mitotic indexes. (**C**)—Density of cells monolayer. (**D**)—Space of surface without cells. *—*p* < 0.05 vs. control, **—*p* < 0.05 vs, NiTi. Data are shown as means ± SEMs.

The influence of BS and composition Ag_2_O NPs·BS on the mammalian cells survival was investigated (Figure 11A). The amount of dead cells on culture plastic (control) was not more than 4%. The same amount of dead cells was observed on BS and composition 0.001% Ag_2_O NPs·BS. Medical alloy nitinol (NiTi) was used as positive control. The amount of dead cells was about 50% higher (~6%) on nitinol. In cell culture on a composition 0.01 or 0.1% Ag_2_O NPs·BS approximately 5.5 or 6.5% of cells were dead, respectively.

The proliferation rate of cells was studied using mitotic index in the logarithmic phase of growth (Figure 11B). The mitotic index in control was 1.2%. Nitinol increased mitotic index up to 1.9%. The mitotic index values on BS and composition Ag_2_O NPs·BS were 0.9–1.3%.

The SH-SY5Y culture density in control was 1.1 × 10^3^ cells/mm^2^ (Figure 11C). The density of SH-SY5Y culture on nitinol increased up to 1.45 × 10^3^ cells/mm^2^. The density of SH-SY5Y cells BS was 1.15 × 10^3^ cells/mm^2^, which comparable with control. The density of SH-SY5Y cells on composition Ag_2_O NPs·BS reached 8.0–1.15 × 10^3^ cells/mm^2^.

Morphological analysis was performed on 3 day of cell growth on studied samples. The space of free surface in control was ~30%. Nitinol decreased free surface to 25%. BS and composition 0.001% Ag_2_O NPs·BS also decreased free surface to 26 and 27%, respectively. Therefore 0.001% Ag_2_O NPs·BS is suitable for cell adhesion and spreading (Figure 11D). Compositions 0.01% Ag_2_O NPs·BS and 0.01% Ag_2_O NPs·BS did not affect space of free surface.

The rheological behavior of synthesized compositions with silver oxide nanoparticles is mainly depends on the polymer matrix medium. Therefore, they may be easily adjusted for various tasks at the synthesis stage.

The generation of high amount of ROS can limit application of NPs of Me_x_O_y_/Me in medicine [52]. ROS production is a main mechanism of Me_x_O_y_/Me NPs cytotoxicity [53], but potential employment of Me_x_O_y_ NPs in wounds healing [54,55] and cancer therapy [56,57,58,59,60,61] is discussed. In present work BS itself did not induce production of ROS. The addition of Ag_2_O NPs in BS significantly increased ROS production. Supplementation of ≥0.1%, Ag_2_O NPs increased H_2_O_2_ production on 700%, and OH-radicals production on 300%. The key required property of Me_x_O_y_/Me NPs composites is balanced cytotoxicity. Me_x_O_y_/Me NPs·polymer composite must kill bacteria, but not act on mammalian cells. It was shown that the Ag_2_O NPs have a more pronounced antibacterial effect against *Escherichia coli* than solid Ag_2_O [62]. Our data on the high antibacterial action of the composition Ag_2_O NPs·BS are in agreement with literature data. A good marker of oxidative stress of cells and oxidation of proteins and DNA are levels of LRPS and 8-oxoGua, respectively [63,64,65]. We observed that BS does not change the production of LRPS and 8-oxoGua. At a concentration of silver oxide nanoparticles of 0.1 wt%, the concentrations of LRPS and 8-oxoGua increased by 2–2.5 times compared to pure BS, approximately 4 molecules of 8-oxoGua per hundred thousand base pairs are formed. This roughly corresponds to the effect of 5 Gy dose of ionizing radiation (radiation-chemical yield 0.78 molecules/100 eV). It is known that 4 Gy is absolutely a lethal dose for humans.

Our data about the level of cytotoxicity of Ag_2_O NPs are lower than literature data in 10–20 times [66,67,68]. We proposed that the difference between our and literature data are caused by differences in applied NPs concentration. We used lesser doses of Ag_2_O NPs.

The BS matrix itself decreased bacterial adsorption to substrate by in 10 times compared with control. The addition of 0.1 Ag_2_O NPs decreases the count of bacteria in culture medium in 33 times and decreased the bacterial adhesion in 2.5 times. In other works, on the addition of silver to various polymer bases, particles of Ag [69,70,71], Ag_2_O [70,72,73], and AgNO_3_ [70] are usually used. In literature NPs concentrations often are more than 1% [70,72], wherein the antibacterial activity of the obtained compositions is less than that of Ag_2_O NPs·BS [73]. Such composites have high antibacterial action against both Gram-positive and Gram-negative bacteria, for example strains of *Klebsiella, Listeria*, *Staphylococcus* and *Bacillus* [74,75,76,77,78].

Ag^+^ cations are known to replace other important metal cations, such as Zn^2+^ and Ca^2+^ in active centers of enzymes, disrupt electron transport chain and oxidative phosphorylation, which cause cell death. In addition, it was suggested that silver ions released by NPs could facilitate the interaction with phosphorous DNA fragments, causing inactivation of DNA replication [52,79]. In present study the effect of the Ag_2_O NPs·BS composite on mammalian cells viability and proliferation (evaluated by mitotic index) were researched. BS itself did not influence on cell viability and proliferation. The supplementation of 0.1 Ag_2_O NPs into BS increased the amount of dead cells in 50% and decreased mitotic index in 30%. Obtained data are in agreement with the literature data on the cytotoxicity of Me_x_O_y_ NPs [80,81,82,83,84,85,86,87,88]. To evaluate the created materials, readers are invited to compare the properties of our material and the closest analogues created earlier (Table 1).

Thus, a promising material based on BS and Ag_2_O NPs has been developed. Obtained material exhibits significant bacteriostatic properties and does not exhibit acute toxicity towards eukaryotic cells. Further directions of research of these composite materials can go in the following directions: (1) Development of composite materials that significantly change the mechanical properties in the transition from the temperature of the human body to room temperature. (2) Investigation of the mechanical properties of materials during long-term operation. (3) Investigation of the possibility of composition regeneration. (4) Evaluate of the influence of nanoparticle size on the main features of the composition. (5) Study of clustering and aggregation of silver oxide nanoparticles with each other and with biological material. (6) Development of compositions with an ordered structure. (7) Investigation of the antibacterial properties of composite materials, including on different types of bacteria. (8) Investigation of the possibility of using a composite material in the fight against biofilms. (9) Research on the long-term effects of compositions on eukaryotic cells. (10) Investigation of the possibility of using the composition in biomedical research, agricultural research, environmental work, the use of the material in the manufacture of clothing and packaging.

## 4. Conclusions

A borosiloxane-based composition with silver oxide nanoparticles has been synthesized and characterized for further biomedical applications. The polymer matrix is chosen in such a way that it does not affect the growth and viability of both bacteria and mammalian cells. The resulting composition with silver oxide nanoparticles has strong bacteriostatic properties with respect to *E. coli* culture, but low cytotoxicity with respect to mammalian cells. The rheological behavior of the composition at low dopant contents is determined mainly by the polymer matrix and is strictly controlled at the synthesis stage. The rheological features of borosiloxane increase the detachment of bacteria from the substrate by one order of magnitude, and the addition of 0.1 wt% Ag_2_O nanoparticles reduces the density of bacterial cultures by 3 times and increases the detachment of bacteria by another 2.5 times. The resulting polymer composition with Ag_2_O nanoparticles has great potential in the field of hyenas and dry disinfectants.

## Figures and Tables

**Table 1:** Comparison of physical and biological properties of previously manufactured nanocomposites based on polymers and nanoparticles containing silver.

Materials	NPs Size, nm	Bacterial Strains	Effect *	MIC/MBC	Results	R **
Chitosan + Ag_2_O NPs	300	*S. aureus*, *E. coli*	BS	–	coating resistant to external influences; antibacterial effect	[89]
Chitosan/ PVP + Ag_2_O NPs	10–30	*S. aureus*, *E. coli*	BS	–	the prepared film has more wound healing property	[90]
Chitosan + Ag_2_O NPs, prepared under various conditions	43–55	*S. aureus*	BS, BC	MIC 4.5 ± 1.5 µg/mL, MBC 19 ± 3 µg/mL for UCLA 8076; MIC 6 ± 0 µg/mL, MBC 22 ± 0 µg/mL, for 1190R	nanocomposites exhibit higher antibacterial activity than any component acting alone	[91]
Polyester (PES) and polyamide (PA) fibers + Ag NPs	10	*S. aureus*, *E. coli*	BS	–	corona-treated PES and PA fibers supplemented by silver NPs exhibited more pronounced antibacterial effect in comparison with fibers without NPs	[92]
Polyurethane (PU) + Ag NPs	∼5	*B. subtilis* *E. coli*	BS	–	PU–Ag 30 ppm had the maximum bacteriostatic effect	[93]
		*S. aureus*, *B. subtilis*, *E. coli*, *P. aeruginosa*, *K. pneumoniae*				
Epoxy/ clay composite + Ag_2_O NPs	5–20	*S. aureus* (ATCC11632); *B. subtilis* (ATCC11774); *E. coli* (MTCC40); *P. aeruginosa* (MTCC7814); *K. pneumoniae* (ATCC10031)	BS	–	Antibacterial activity against all tested strains	[94]
Ag-doped poly (ε-caprolactone) (PCL) fibers	-	*E. coli*, *P. aeruginosa*	BS	–	antibacterial activities against *E. coli* of the all composites were equal (nearly 100), the antibacterial activity against *P. aeruginosa* depended on spinning temperature	[95]
poly-N-isopropylacrylamide (pNIPAM) + Ag NPs	1–10	*E. coli*, *S. aureus*	BS	–	significant bacteriostatic activity against gram-negative *E. coli* and gram-positive *S. aureus*, depending on the size of nanoparticles and the amount of AgNO 3 used in the synthesis	[96]
Chitosan + Ag NPs	1–50	*E. faecalis*, *S. typhimurium*, *L. monocytogenes*, *P. aeruginosa*	BS	MIC 1.56 µg/mL for *E. faecalis*; 3.125 µg/mL, for *S. typhimurium*; 7.8 µg/mL, for *L. monocytogenes*; 12.5 µg/mL, for *P. aeruginosa*	nanocomposites exhibited bacteriostatic activity, but did not exhibit antifungal activity	[97]
composite resins containing 1% Ag_2_O and ZnO NPs	20	*S. mutans*, *Lactobacillus* sp.	BS	–	composite resins containing zinc oxide and silver nanoparticles can significantly inhibit the growth of two important microorganisms in the oral cavity: *Streptococcus mutans* and *Lactobacillus*	[98]

*—BS—bacteriostatic, BC—bactericidal; **—R—references.

## Data Availability

The data used to support the findings of this study are available from the corresponding author upon request.
